# Social Prescribing Competence among Community Pharmacists and Pharmacy Students in Norway

**DOI:** 10.3390/pharmacy12020043

**Published:** 2024-03-01

**Authors:** Riyaan Mahamud Gabeyre, Misbah Hussein, Siedra Salih, Salia Amir, Parisa Gazerani

**Affiliations:** 1 Department of Life Sciences and Health, Faculty of Health Sciences, Oslo Metropolitan University, 0130 Oslo, Norway; 2 Department of Health Science and Technology, Faculty of Medicine, Aalborg University, 9260 Gistrup, Denmark

**Keywords:** social prescribing, community referral, non-clinical intervention, community pharmacist, pharmacy student(s), competence, Norway

## Abstract

**Background and aim:** Social prescribing, which links patients to non-clinical services and involves general physicians, has been gaining traction. Community pharmacists, who are integral to primary healthcare, have untapped potential in social prescribing. This study explores social prescribing competence among Norwegian community pharmacists and pharmacy students. **Method:** A cross-sectional study utilizing an anonymous online questionnaire to collect quantitative data was conducted. Inspired by the limited relevant literature, the questionnaire was constructed, pilot-tested, and distributed in a one-week window within a Facebook group for Norwegian pharmacists. The questionnaire comprised 23 questions categorized into demographic details and competence assessment, covering general knowledge, attitude, and barriers/facilitators related to social prescribing. Statistical analyses were employed to determine the competence of the participants. **Results:** The online questionnaire collected data from 96 participants, primarily females (79.2%), aged 25–34 (40.6%), who were identified as community pharmacists (49.0%). Most (91.7%) worked in community pharmacies, with 31.3% having over 10 years of experience. Despite positive client relationships (93.8%), statistical analysis revealed no significant associations between competence and variables such as work experience, education, or gender. The custom scoring system yielded an average competence score of 1.98 on a 5-point scale, with attitudes and perceptions of participants scoring 3.82. Overall competence was calculated at 3.4, indicating a moderate level. **Conclusions:** The findings of this study reveal that the participants had limited knowledge regarding social prescribing, emphasizing the need for education. However, the participants showed strong enthusiasm for competence development. This groundwork paves the way for future investigations centered on pilot-testing strategies to boost social prescribing knowledge and engagement among Norwegian community pharmacists and pharmacy students.

## 1. Introduction

### 1.1. Social Prescribing or Community Referral

Social prescribing, or community referral [1], is a process facilitated by healthcare professionals that directs patients to diverse societal services, typically overseen by a social prescription coordinator [2]. The coordinators, in consultation with the patient, assess their needs to create a personalized plan and connect them to non-clinical services [3], such as support groups, activity clubs, courses, or educational venues supported by municipalities or voluntary organizations [2]. Social prescribing, which emerged in the early 21st century, aims to enhance health and well-being beyond established clinical strategies [4] and can be seen as a holistic approach to health and wellness. At its core, social prescribing involves healthcare professionals “prescribing” non-medical interventions to patients in order to address their social, emotional, and practical needs [5]. These interventions typically include activities such as arts and creative therapies, exercise programs, community support groups, volunteering opportunities, and access to social services [6]. The mechanism through which social prescribing is thought to work is by recognizing that health and well-being are influenced by myriad factors beyond just medical treatment [2]. By addressing social determinants of health such as loneliness, poverty, lack of access to community resources, and mental health issues, social prescribing aims to improve overall health outcomes and quality of life [7]. Through tailored interventions, patients are empowered to take control of their own health and well-being, leading to a more holistic approach to healthcare [8]. This approach, in theory, must benefit any society around the globe; however, the format and style may differ to fit the needs of the society, culture, and healthcare system. Generally, social prescribing initiatives have been hypothesized to provide numerous health and wellness benefits, including reductions in symptoms of anxiety and depression, improved self-esteem and confidence, enhanced social connections and support networks, better management of chronic conditions, increased physical activity levels, and overall improved quality of life. By fostering a sense of belonging and purpose within communities, social prescribing not only addresses individual health needs but also strengthens community resilience and cohesion [9].

In theory, social prescribing has potential benefits for various patient groups, aligning with the UN’s sustainability goals for health and quality of life [10]. The World Health Organization’s definition of health as complete well-being resonates with the inclusive nature of social prescription [10]. Community referral, in alignment with this holistic perspective, aids in identifying psychosocial and socio-economic challenges, making it crucial for optimal patient referrals [11]. Social prescription is particularly impactful for vulnerable groups, such as the elderly and children, those with physical/psychological disorders, and excessive health service users, as it offers non-drug-based services that foster social interactions, promote mental health, and enhance physical activity [12,13]. In many cases, social prescriptions complement treatment plans, contributing to mental health prevention and overall well-being, as evidenced in a 2023 meta-analysis demonstrating positive outcomes such as increased self-image and improved mental well-being [14]. Despite the limited availability of relevant studies in the literature [2,11,12,13], a meta-analysis [14] indicated that social prescription coordinators could offer more effective assistance than general practitioners or other health personnel. Coordinators, through in-depth exploration of the needs of patients and extended interaction times, were perceived as attentive and understanding, providing tailored services that precisely addressed the needs of individuals [14].

In England’s NHS, the Bromley by Bow Center (founded in 1984) exemplifies community-driven services, from education to nature-based offerings [15]. Recent attention to social prescribing, particularly involving general physicians, has expanded the adoption of non-clinical services [15]. These services, which are independent of clinical offerings, include art therapy, physical activity, book therapy, or ecotherapy (involving nature walks), serving to promote the holistic health of patients [16]. Social prescriptions tailored to the needs of the patient enhance understanding and motivation and foster a positive mood, improving both physical and psychological health conditions [17]. Although social prescribing has gained attention, it is not uniformly distributed globally, suggesting an evolving landscape in primary care that integrates social aspects for a comprehensive approach to health [2,4,15,18].

### 1.2. Role of Pharmacists in Social Prescribing or Community Referral

Pharmacists, who have long been pivotal in public health, fulfill essential roles in terms of medicine expertise and dispensing, ensuring the proper use of medicine [19]. Pharmacy education emphasizes the acquisition of both practical and theoretical competence [20]. Evolving alongside healthcare automation, pharmacists have come to play a more prominent role in primary healthcare, offering services such as inhalation guidance, medication initiation, and vaccinations [21]. Community pharmacists contribute to primary health services, fostering trust through direct customer interactions, making them ideal for the dissemination of low-threshold health- and medicine-related information [22,23]. According to “apotekstatistikk”, with 58.5 million visits in 2022 in Norway, community pharmacies serve a diverse clientele, including those seeking prescriptions, over-the-counter medicines, consultations, or vaccinations. Despite being accessible to patients of all ages and needs, public awareness of available services is lacking, and some community pharmacists remain unaware of their potential role in social prescribing [24]. Cross-sectional studies in England, Scotland, and Wales have highlighted the limited involvement and knowledge of pharmacists in community referrals, emphasizing the need for research in this area [24]. A systematic literature search has revealed the lack of data on the roles of pharmacists in social prescribing around the globe, emphasizing the necessity for further research to guide the establishment of social pharmacy among community pharmacists, enrich the profession, and enhance patient and public health [25]. The limited literature on the topic further underscores this gap, with no identified studies in Norway. The only record we found was an initiative called Red Cross’s “Social Recipe” in Bergen, Norway [26], delivered through general physician-issued social prescriptions. We believe that the healthcare system in Norway and community pharmacists have the capacity to accommodate social prescribing efficiently. In Norway, the concept of “prescribing” extends beyond the prescription of traditional medication to encompass a broader spectrum of healthcare interventions. While physicians play a central role in diagnosing and treating medical conditions, Norwegian healthcare professionals—including pharmacists—are increasingly involved in promoting holistic health and wellness. Pharmacists in Norway are not only dispensers of medications but are also integral members of the healthcare team, providing counseling, medication reviews, and health promotion services. Additionally, Norwegian pharmacies often serve as hubs for health education and community outreach programs, in alignment with the principles of social prescribing. Norway’s robust social support system further complements its healthcare delivery model. The country prioritizes social welfare, with extensive public services aimed at supporting individuals across various life domains, including healthcare, education, employment, and social inclusion. This comprehensive approach to social support aligns well with the philosophy of social prescribing, facilitating the seamless integration of non-medical interventions into primary care settings. In the context of health and wellness, Norway emphasizes preventive care and patient empowerment, with a focus on promoting healthy lifestyles and fostering self-management skills among its population. Primary care services are widely accessible, with an emphasis on continuity of care and interdisciplinary collaboration to address the diverse needs of patients. Overall, Norway’s healthcare system, with its inclusive approach to healthcare delivery and strong social support networks, provides an ideal environment for the implementation and success of social prescribing initiatives, contributing to improved health outcomes and enhanced well-being for its population.

Hence, this exploratory study takes the initial step to assess community referral competence among community pharmacists and pharmacy students in Norway. We excluded clinical pharmacists at hospitals, pharmacists employed at pharmaceutical companies and pharmaceutical organizations, and those who were administrative staff and policymakers. The findings are expected to serve as a foundational platform for subsequent research, elevating the theme’s significance in the country. Moreover, the results provide insights into the establishment of such a system in Norway, emphasizing the necessity of collaborative competence development among healthcare providers. We hypothesize that building competence is the crucial and fundamental first step toward effectively implementing social prescribing. Pharmacists in Norway must undergo comprehensive education and training that equip them to understand and address the social determinants of health. Their educational curriculum must include coursework on public health, health promotion, and social determinants of health, providing them with a solid foundation to recognize and respond to the broader factors influencing patient well-being beyond just medications. Patients in Norway may expect community pharmacists to offer more than just medication dispensing services, similar to other countries [27]. They often seek pharmacists for advice on self-care, minor ailments, and medication management. There is a growing recognition among patients of the roles that pharmacists can play in promoting holistic health and well-being. Physicians and other healthcare professionals increasingly view pharmacists as valuable members of the healthcare team and expect pharmacists to collaborate closely with them, providing medication expertise, conducting medication reviews, and offering patient counseling. The potential for pharmacists to contribute to broader health initiatives has been acknowledged [28], and social prescribing may be considered a fundamentally valuable initiative. Interestingly, the Norwegian government recognizes pharmacists as key stakeholders in the healthcare system and expects them to contribute to public health objectives, including promoting health literacy, medication adherence, and preventive care. These points emphasize the importance of maximizing the role of pharmacists within the primary care setting in order to optimize healthcare delivery and outcomes. We based our study on these observations and the fact that, in Norway, various health promotion services, counseling, and preventive care programs are constantly being introduced and offered. Social services agencies also provide support for vulnerable populations, including the elderly, low-income individuals, and those with disabilities. Various voluntary organizations and community groups also actively work to foster social connections and provide assistance in times of need. To build a more efficient link between these various elements in Norway, we argue that involving pharmacists in social prescribing’s loop is uniquely valuable, based on their unique accessibility and expertise. Pharmacists possess medication expertise and have established relationships with patients, making them well-positioned to identify social needs and facilitate appropriate referrals or interventions. While other healthcare professionals and community organizations play important roles in social prescribing, the involvement of pharmacists can complement existing efforts and ensure a more holistic approach to patient care. Therefore, our study can also be seen as an advocacy factor for the involvement of pharmacists in social prescribing, aligning with the broader goal of leveraging all available resources to address the social determinants of health and promote comprehensive well-being in the Norwegian community and beyond.

## 2. Methods

### 2.1. Study Design and Population

We employed a cross-sectional study design, utilizing an anonymous online questionnaire targeted at community pharmacists and pharmacy students in Norway. This approach aimed to capture a snapshot of competence in terms of social prescribing, providing a descriptive overview of measurable data with high efficiency and broad participation at minimal cost.

### 2.2. Construction of the Questionnaire 

In the absence of a gold standard or validated questionnaire for assessing competence in social prescribing among community pharmacists and pharmacy students, we drew inspiration from the limited literature to construct our questionnaire. Inspired by the study of Taylor et al. [24], a few questions were adopted and included, as follows: Are you familiar with the term social prescribing? (question 10); Have you used social prescribing in your working day? (question 12); How confident are you that you can identify people who can benefit from social prescribing (community referral)? (question 19); If you are to become more involved in social prescribing, what kind of training do you think you need to be able to perform this role? (question 21); Could you imagine having greater responsibility for the social prescribing (community referral) of patients as a pharmacist? (question 15)

The online anonymous questionnaire, developed in Norwegian using Nettskjema (Supplementary Materials), comprised purposeful questions, with a majority being closed-type questions offering pre-defined choices. Open boxes were included in several questions, allowing participants to share their opinions. The questionnaire was introduced with information about the research group, the study purpose, and expectations, and was divided into two main parts. The first part gathered demographic information through nine short multiple-choice questions, while the second part had two sub-sections: the first gauging general knowledge of social prescribing with five questions, and the second exploring personal views on facilitators and barriers. The entire questionnaire included 23 questions, combining closed, open, and Likert scale types (1 = strongly disagree, 2 = disagree, 3 = neither agree nor disagree, 4 = agree, 5 = strongly agree), and took approximately 5–7 min to complete. Questions with an “other” option provided a space for respondents to provide free-text answers, if desired.

### 2.3. Pilot Testing of the Questionnaire

The initial questionnaire was distributed to a pilot group of six individuals, including those with and without a pharmaceutical background. This pilot test aimed to identify potentially unclear questions, assess the answer options, and gather scientific feedback from those with a pharmacy background. Participants were given 24 h to respond, providing an opportunity for in-depth assessment and feedback. The average time spent on the questionnaire, based on reflections and valuable comments from the pilot group, guided adjustments and further clarification. The obtained feedback prompted the re-formulation of some questions, alternative answers, and a re-ordering of questions to improve the overall flow of the questionnaire.

### 2.4. Ethical Assessment

Given the study’s design and content, no ethical or personal data handling approval was required. The questionnaire’s introduction explicitly stated that the collected data would only be used for academic purposes. The questionnaire was entirely anonymous, and participation was voluntary. While explicit consent for data use was not sought, participants could provide implicit consent by participating in the questionnaire. Demographic information collected included gender, age, educational institution, and district, aimed at forming an overview of the target group. As the questionnaire was population-based, no sensitive personal information identifying participants was collected.

### 2.5. Data Collection and Handling

The Norwegian web-based questionnaire was delivered through the Nettskjema tool, with the link being shared on the valid and professional Facebook group for pharmacists and made available for 7 days. Upon closing the questionnaire in November 2023, raw data were automatically extracted and transferred to Microsoft Excel for descriptive and statistical analyses. Part 2 of the questionnaire included Likert scale questions (1–5), allowing for the calculation of scores for each participant. A self-defined “competence score” was computed by averaging points from questions 11, 18, and 19, which captured knowledge, skills, abilities, and attitudes related to their competence. This method aligned with the study’s aim to assess both knowledge and attitudes. To differentiate between answer options, a point-interval system was implemented for the competence score (Table 1). It is important to note that competency is indeed multifaceted, but at its core, it encompasses a blend of practical and theoretical knowledge, cognitive skills, behaviors, and values necessary to effectively perform a given task or fulfill a particular role. This definition acknowledges the dynamic nature of competency while highlighting its fundamental components essential for successful performance in various contexts.

### 2.6. Statistical Analysis

First, the normal distribution of data was tested to guide the application of parametric or non-parametric methods. We also defined the study variables as dependent variables (i.e., those that are supposed to be investigated) and independent variables (i.e., those that affect the dependent variables). These variables were further divided into categorical or continuous variables. Table 2 provides our null hypotheses, variables, and the statistical tests used for testing the hypotheses. The significance level was set at 0.05.

## 3. Results

### 3.1. Socio-Demographic Information

The questionnaire, conducted between 17 and 23 November 2023, received complete responses from 96 participants. Socio-demographic information was gathered using part 1 of the questionnaire (Table 3).

### 3.2. Questions with Dichotomous Answer Options

**Figure 1:**
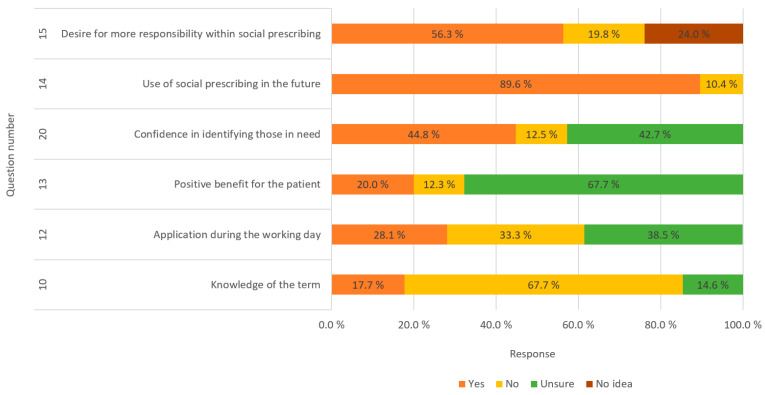
Overview of answers to the questions with dichotomous answer options.

Part 2 of the questionnaire comprised questions with dichotomous answer options, including “no”, “yes”, or a third response option, which could include variations such as “not sure”, “no idea”, or “do not want to answer”. Figure 1 provides an overview of the items, along with the corresponding percentage distribution of responses.

Follow-up questions (see the questionnaire in the Supplementary Materials) were posed to understand why respondents could not envision greater responsibility, receiving responses from 33 individuals. Reasons included pharmacists having numerous other tasks (33%), insufficient knowledge about the topic (46%), and difficulty identifying patient needs (6%), while 15% cited other reasons. Regarding why respondents desired greater responsibility, 59 participants provided insights. Motivations included reducing the burden on other health sectors (12%), facilitating help for patients (51%), and offering a low-threshold service (32%), while 5% mentioned other reasons.

An examination of the agreement of participants regarding the usefulness of pharmaceutical involvement in community referrals revealed that, of the 96 respondents, 11.5% strongly agreed, 60.4% agreed, 2.1% disagreed, and 26% were neutral. 

**Figure 2:**
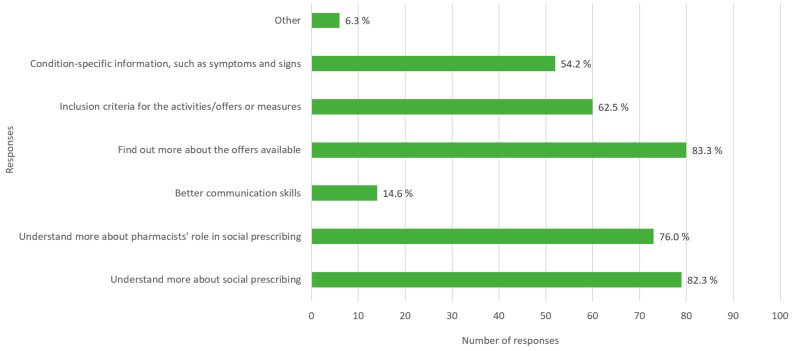
Distribution of preferred training for increased involvement in social prescribing as perceived by respondents.

When inquiring whether the training pharmacists wished for increased involvement in community referrals, 364 responses were received (please note that more than one choice among alternative responses was allowed here). Of the 96 respondents, 82.3% expressed a need to “understand more about social prescribing (community referral)”, while 76% indicated a desire to “understand more about the pharmacist’s role in social prescribing (community referral)”. See Figure 2 for detailed results.

We investigated the confidence of pharmacists in identifying individuals who might benefit from community referrals. Of the 96 respondents, 49% were uncertain about their ability, with “neither sure nor unsure” being their response. Meanwhile, 26% expressed confidence, 12.5% felt insecure, 6.3% were very insecure, and another 6.3% were very confident.

Examining the familiarity of respondents with community referrals from the past, the results showed that 41.7% had very poor knowledge of social prescribing, 27% had poor knowledge, 8.3% considered their knowledge to be “good,” and 23% remained neutral on the topic.

**Figure 3:**
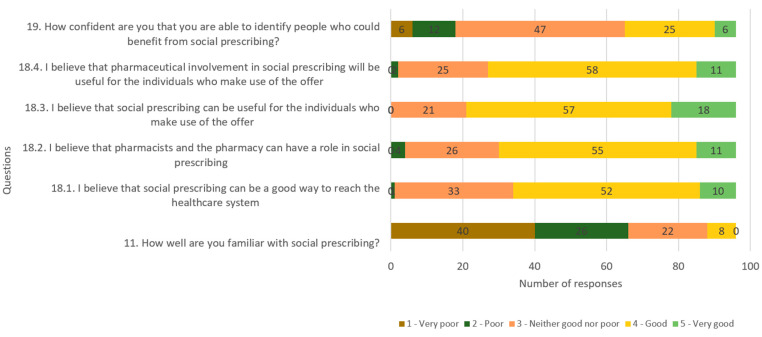
Overall competence of candidates as assessed by competence questions.

The overall competence of the candidates is illustrated in Figure 3.

The average overall competence score of the participants was 3.4 out of 5, categorized as “medium” according to the pre-determined values (see Table 1). The average score for the perception of social referral was 3.8 out of 5, indicating a “good” level. However, the average score for knowledge within social referral was 1.98 out of 5. For the analysis, parametric tests were employed, considering the identification of normally distributed data. 

The competence questions and their identified average scores are listed in Table 4. These questions were integral to assessing the perceptions, beliefs, and confidence of participants related to social prescribing and the involvement of pharmacists.

**Figure 4:**
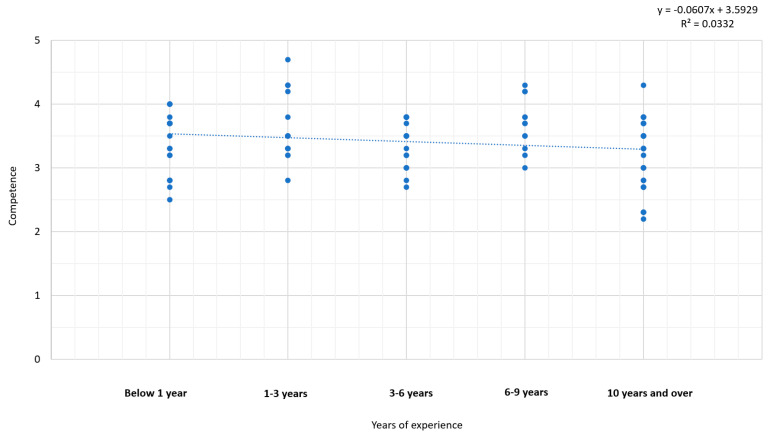
Relationship between the variables of experience and competence.

The results from the regression analysis (Figure 4) revealed no significant relationship between years of work experience and competence. The R^2^ value of 0.0332, along with a confidence interval ranging from −0.1278 to 0.0064, suggests that there is no discernible relationship between the two variables under investigation.

An analysis of variance was employed to investigate whether there was a significant difference in competence within community referral between community pharmacists and pharmacy students. The results indicated no significant difference across different educational levels in terms of competence (*p* = 0.4079). An independent *t*-test was conducted to assess whether a significant difference in competence within community referral existed between genders. The outcome demonstrated no significant difference between men and women regarding competence within social referral (*p* = 0.3284). A summary of these results is provided in Table 5.

## 4. Discussion

To the best of our knowledge and based on the available literature, this research marks the inaugural exploration of its kind in Norway. A total of 96 pharmacists and pharmacy students willingly and anonymously participated in our questionnaire-based investigation. Despite the study’s inherent limitations in terms of size and its exploratory nature, we provide a discussion of the findings below.

### 4.1. Knowledge and Perceptions towards Social Prescribing among Pharmacy Students and Community Pharmacists in Norway

A total of 67.7% of participants indicated their unfamiliarity with the concept of social prescribing, indicating its relative obscurity and under-utilization in Norway. Notably, there is an absence of any formal framework or organization dedicated to implementing or coordinating social prescribing within the healthcare system in Norway. This void emphasizes the urgent requirement for advocacy, awareness, and educational initiatives seamlessly woven into pharmacy programs or provided as continuous education for community pharmacists, acknowledging their pivotal role as primary healthcare professionals.

Addressing this informational gap can take diverse forms, ranging from seminars, formal lectures, themed evenings, and round-table discussions to the provision of self-education materials for digital courses. Given that the term originates from studies in England, Scotland, and Wales [24], it is imperative to accurately translate and define it within the Norwegian context. The absence of a well-defined Norwegian equivalent for “community referral” or “social prescribing” may contribute to the overall lack of familiarity among respondents. Indeed, the questionnaire revealed that 28.1% of respondents believed they had incorporated social referrals into their daily responsibilities, while 38.5% remained uncertain. This underscores the pervasive ambiguity surrounding the term, suggesting that some pharmacists might be engaged in such practices unknowingly. In essence, while there is a recognized deficit in understanding, a noteworthy portion of pharmacists still perceived themselves—to varying extents—as inadvertently integrating social prescribing into their professional routines. A notable number (44.8%) of respondents conveyed confidence in their ability to identify patients who could benefit from non-clinical services. This confidence likely stems from a strong emphasis on fostering excellent customer relations and honing communication skills, an integral aspect of daily life in community pharmacies, particularly in terms of discerning the needs of customers. Crucially, the results revealed a substantial willingness among respondents to incorporate social prescribing or community referrals into their daily services at community pharmacies. A resounding 89.6% expressed interest in exploring various forms of social referral. The positive inclination among pharmacy students and community pharmacists in Norway observed in this study signifies a readiness to embrace the concept, suggesting a growing willingness to incorporate this responsibility into their daily professional tasks. This is in alignment with the findings reported in the studies of Taylor et al. and Lindsey et al. [25], where, despite a noted lack of knowledge, pharmacists displayed a high level of commitment to and interest in actively participating in social prescribing initiatives.

The obtained findings revealed a limited awareness of the social prescribing concept among respondents, with only 17.7% demonstrating familiarity and a substantial 41.7% exhibiting a notably deficient understanding of the subject. In particular, only 8.3% opted for the response indicating a “good” level of knowledge. This collective data underscores a noteworthy disparity, indicating that, while pharmacists possess commendable proficiency in clinical services and offerings, they trail behind in their grasp of non-clinical services. Moreover, an examination of the attitudes of participants regarding the utility of community referral revealed that a substantial 60.4% agreed on the beneficial impact of the involvement of community pharmacists in community referral. Conversely, only 2.1% disagreed with this perspective. This suggests a prevailing acknowledgment among pharmacists regarding the potential efficacy of community referral, highlighting a positive inclination toward such engagements. In the study of Taylor et al. [24], who investigated the experiences and attitudes of pharmacists with respect to social prescribing in England, Scotland, and Wales, 36.7% of the participants had prior knowledge of social referral, which is higher compared to the 17.7% observed in our study. Despite these differences, both studies revealed a positive attitude towards social referral among the participants, with percentages of 83.8% [24] and 71.9% (the current study). In the study of Taylor et al. [24], 55.9% of the candidates expressed either high or moderate confidence in identifying patients who could benefit from community referral; meanwhile, this proportion was slightly lower in our study, at 32.3%. This discrepancy could be attributed to the broader implementation of community referral schemes in the U.K., potentially making it more straightforward for pharmacists to discern their roles within such programs. Interestingly, responses from both questionnaires indicated a shared pattern of limited knowledge about social referral among the participants. However, there was a notable interest in learning more about the topic, coupled with optimism for its future utilization. This suggests a common theme of receptivity to further education and the potential integration of social prescribing practices among pharmacists, regardless of regional differences in the prevalence of such programs.

The design of the questionnaire allowed for the identification of potential obstacles that need to be overcome for pharmacists to assume a more substantial role in community referral. The foremost challenge identified pertains to comprehending the framework and responsibilities of community pharmacists in the context of social prescribing. A notable 82.3% of respondents expressed a need for enhanced knowledge in this area, with an additional 76% expressing a desire for a deeper understanding of the pharmacist’s role. Interestingly, a modest 14.6% highlighted the possibility that improved communication skills could present a barrier to effective community referrals. This suggests general confidence in their existing communication abilities, emphasizing the potential of leveraging these skills to effectively discern the needs of patients.

The results of this study emphasize that communication theory and the handling of diverse patient groups are integral components of pharmacy education [22], providing fully qualified pharmacists with a solid foundation in these areas. Furthermore, the questionnaire indicated a prevailing aspiration among pharmacists to assume greater responsibilities within the health service [29], recognizing pharmacies as accessible and low-threshold services. The expanding responsibilities of pharmacies reflect an acknowledgment of their evolving role in healthcare [19,30]. Pharmacists, according to the results, expressed a positive inclination towards increased involvement in community referral. While physicians bear the primary responsibility for diagnosing and arranging medical treatment—including potential community referrals—pharmacists can potentially offer a unique perspective. Their expertise extends beyond drug treatments, encompassing insights into the mechanisms of action, side effects, and efficacy of drugs. This distinctive viewpoint creates an opportunity to explore non-clinical alternatives or complementary approaches to drug treatment. The questionnaire’s results underscore the belief among respondents that pharmacists should acquire a more profound understanding of social referral to effectively fulfill this expanded role.

At present, Norway lacks an implemented system enabling pharmacists to refer patients to diverse non-clinical services within the primary healthcare setting. The establishment of such a system requires a multi-faceted approach, with two pivotal steps being the cultivation of awareness, knowledge, and training regarding existing or prospective activities/offers, as well as the formulation of inclusion criteria tailored to the individual location and needs of the patient. Additionally, it is imperative to institute a comprehensive recording mechanism for referrals in order to document their efficacy over time. These propositions are rooted in the study’s findings, which revealed that only 26% of respondents felt sufficiently confident to identify patients who could benefit from community referral. This hesitancy may stem from various factors, including the perceived time-consuming nature of identifying patient needs. In the long term, a plausible scenario involves pharmacists specializing in the field of social prescribing. Leveraging evolving technology and automation can play a pivotal role in streamlining tasks, thereby allowing pharmacists to dedicate more time to specialized responsibilities, such as community referrals tailored to individual patient needs.

Contrary to the prevailing stereotype of a pharmacy transaction characterized by simple exchanges and dialogues, a transformative cultural shift is essential. There is a pressing need to elevate awareness regarding the integral role of pharmacists in society. By fostering this awareness, pharmacies can transcend the image of mere retail spaces, emerging as vital contributors to primary healthcare services. This shift positions pharmacists as experts who are capable of offering a diverse range of tasks and services, thereby underscoring their competence and significance within the broader healthcare landscape.

### 4.2. Competence

In this study, the term “competence” serves as a comprehensive umbrella, encompassing both knowledge and attitudes. The average competence score of respondents was calculated as 3.4 out of 5, indicative of a “medium” level of competence. This composite score is derived from a relatively modest average of 1.98 in general knowledge. Meanwhile, perceptions and attitudes exhibited more positivity, with an average score of 3.82 corresponding to a “high” level. A strategic decision was made in this study to treat competence as a continuous variable. This approach facilitates the use of linear regression as a statistical analysis method, leveraging the linear relationship between variables. Alternatively, analyzing competence as a categorical variable could have been pursued, leading to the application of the chi-square test as a suitable statistical analysis. It is worth noting that, while linear regression provides insights into the nature and strength of relationships between variables, the chi-square test is focused on revealing differences between distinct groups. This methodological choice reflects the nuanced understanding sought in examining the multi-faceted concept of competence within the context of this study.

The linear regression analysis did not reveal a significant association between the experience of pharmacists and their competence in community referral. Consequently, we cannot definitively conclude whether a connection exists between these variables or not, acknowledging that the study did not encompass all pharmacists and pharmacy students in Norway. The possibility of a type 2 error remains, emphasizing the need for caution in drawing conclusive statements.

When exploring the impact of gender on competence within community referral among pharmacists, an independent *t*-test with a *p*-value of 0.328 indicated no discernible difference. Notably, there was a general lack of awareness regarding community referrals across genders. However, it is essential to recognize a potential weakness in the analysis stemming from a notable gender imbalance among questionnaire participants, with nearly 80% being women. This disparity could introduce bias, given that pharmacy is predominantly a female-dominated profession in Norway and other places [31]. Future investigations should aim for a more balanced representation to ensure a comprehensive understanding.

Moreover, an analysis of the potential influence of education level on competence in social prescribing revealed no significant difference between pharmacy students and community pharmacists. This outcome may be interpreted as being due to the fact that community referral is not part of the study plan or training curriculum in pharmacy programs, resulting in a similarity in competence levels. Nevertheless, it is crucial to note that the study results were based solely on the 96 respondents, necessitating caution when generalizing findings to the broader population of pharmacists and pharmacy students throughout Norway. Further research with a more extensive and diverse sample is recommended for a more comprehensive understanding of these dynamics.

### 4.3. Study Strengths and Limitations

The literature review revealed limited research on the involvement of pharmacists in community referral, with most studies originating from Great Britain, particularly focusing on social referral. Notably, a comparable cross-sectional study has been conducted in England, Scotland, and Wales [24]. For the first time, this study explored the theme in Norway.

Utilizing a quantitative cross-sectional design was considered suitable for obtaining a snapshot of the knowledge and perceptions of pharmacy students and community pharmacists regarding social prescribing. However, this approach has inherent limitations and biases. Although more robust study designs, such as case–control or cohort studies, could offer deeper evidence, they are resource-intensive. Our questionnaire, which was designed for efficient data collection, allowed us to quickly gather information from a large participant pool. While cost-effective and practical, our self-made questionnaire lacked standardization. A structured framework should be adhered to in order to navigate through the diverse phases of questionnaire development and translation. Employing a range of statistical methods is imperative to ascertain the psychometric soundness of the questionnaires, thereby ensuring a rigorous assessment of their reliability and validity. Therefore, the next logical step must include the construction and validation of a reliable and valid questionnaire to make it a standard one in the field. Ideally, qualitative studies, such as focus group interviews, can also be added in the next steps to gain in-depth insights. Despite being time-consuming and financially demanding, interviews offer a more nuanced understanding.

Our questionnaire, based on a semi-structured questionnaire, included various question types to maintain participant engagement. Anonymization was crucial due to the profession’s gender skew. Despite the response bias towards women, future efforts should aim for a more balanced gender representation for comprehensive understanding. Potential biases—including recall bias—might have influenced the results, especially among those familiar with “Social prescribing”. Understanding these biases is critical for interpreting the study outcomes. Considering validity, internal validity depends on the sample representing the population. With only 96 voluntary participants from a single distribution channel, this study might not be fully representative of all Norwegian community pharmacists and pharmacy students. We expected a minimum of 5 answers per question of the questionnaire (total of 23), where the expected number was 115 respondents. Therefore, with 96, we did not succeed in fulfilling this. However, a recent meta-analysis from 2022 shows that the response rate of online surveys in published research is 44.1% [32]. Considering this average, we have had an acceptable response rate (83.5%). However, an optimal way would be to estimate the target population size and calculate the response rate properly. We are also aware that the short time of the open call for participation (7 days) might influence the response rate and recommend a prolongation of time for accessibility to online questionnaires in future studies. Furthermore, the external validity is compromised, as the findings may not generalize beyond Norway. Additionally, the cross-sectional design provides only a snapshot rather than a comprehensive overview over time.

Generalizing findings from the Norwegian context to pharmacists in other countries can provide valuable insights for global discussions on the implementation of social prescribing [33]. Based on the limited literature and the reported Norwegian experience, we can highlight the importance of expanding the roles of pharmacists beyond traditional medication dispensing in order to encompass broader healthcare services [34], including social prescribing. Pharmacists in other countries could similarly determine their potential, advocate for involvement in social prescribing initiatives, and leverage their accessibility, expertise, and established patient relationships to address social determinants of health [35]. The successful implementation of social prescribing in Norway requires close collaboration between pharmacists, physicians, and other healthcare professionals, emphasizing the importance of interdisciplinary teamwork [36] in delivering holistic care. Pharmacists can benefit from fostering collaborative relationships with healthcare providers [37], community organizations, and social services agencies to effectively address the social needs of patients. Providing pharmacists with education and training on social determinants of health, communication skills, and community resources is crucial for effective social prescribing. Hypothetically, any country can incorporate relevant coursework into their pharmacy curricula and offer continuing education opportunities to ensure that pharmacists are equipped to identify social needs and facilitate appropriate interventions. Government support and policy frameworks that recognize and promote the involvement of pharmacists in social prescribing are essential. Policymakers around the globe can also consider the use of integrated approaches to integrate pharmacists into primary care settings and incentivize their participation in public health initiatives [38]. This may include policy reforms, funding initiatives, and regulatory changes to facilitate their engagement in social prescribing activities. While general principles of social prescribing can be applied globally, it is important to recognize the unique characteristics and healthcare systems of each country [39]. Pharmacists should adapt social prescribing interventions to suit the specific needs, resources, and cultural contexts of their communities. Sharing experiences and best practices internationally can help inform tailored approaches to the implementation of social prescribing. Continued research and evaluation of social prescribing programs are essential to building an evidence base and informing best practices. Pharmacists in other countries can contribute to this knowledge base through conducting research, participating in collaborative studies, and sharing their experiences through publications and professional networks [10,39,40].

## 5. Conclusions and Future Perspectives

The study highlighted a noteworthy gap in the knowledge of community referral among pharmacists, reflected in the overall medium level of self-perceived competence. However, the respondents exhibited a strong commitment to pursuing additional training, indicating a potential avenue for improvement. Intriguingly, our findings revealed that variables such as experience, education level, and gender did not exert significant influences on the competence of the target group, indicating an element of uniformity in their responses. Despite the acknowledged limitations, including a relatively small sample size, the study uncovered positive attitudes among pharmacists and pharmacy students toward the prospective use of social referrals. This suggests the viability of implementing community referral training within both pharmacy practice and academic curricula. Given the dearth of existing information on this subject, there are evident opportunities for further research. Utilizing methodologies such as focus group interviews could provide more insights into the perspectives of pharmacists and pharmacy students. Additionally, there is potential for practical implementation and evaluation through pilot testing of a social prescribing scheme specifically involving community pharmacists in Norway. Assessing the effectiveness of such a program would not only contribute to the body of knowledge in this area but could also offer practical insights into the feasibility and impact of community referral initiatives within the Norwegian context. Pharmacists from various countries can potentially enrich this knowledge repository by engaging in research, joining collaborative studies, and disseminating their insights via publications and professional networks.

## Figures and Tables

**Table 1:** Competence score intervals.

Answer Alternatives	Number of Points	The Score Intervals for Competence
Very high	5	≥4.5
High	4	3.5–4.4
Average	3	2.5–3.4
Low	2	1.5–2.4
Very low	1	≤1.4

**Table 2:** Overview of hypotheses, variables, and statistical analysis.

Hypotheses	Independent Variable	Dependent Variable	Statistical Analysis
H0: There is no significant relationship between competence in social prescribing or community referral and experience in the pharmacy profession	Experience in the pharmacy profession Type: Categorical ordinal	Competence in social referral Type: Continuous interval data	Linear regression
H0: There is no significant difference in social prescribing or community referral competence between pharmacists, provisional pharmacists, and pharmacy students	Education level Type: Categorical (independent groups)	Competence in social referral Type: Continuous interval data	Analysis of variance
H0: There is no significant difference between males and females in social prescribing or community referral competence	Gender Type: Categorical dichotomous	Competence in social referral Type: Continuous interval	Independent t-test (parametric) or Mann–Whitney U test (non-parametric)

**Table 3:** Socio-demographic information of the participants.

Information	Answers	Number of Answers (Out of 96)	Percentage (%)
Gender	Woman	76	79.2
	Man	20	22.8
	Other	0	0
	Do not want to answer	0	0
Age	18–24	22	22.9
	25–34	39	40.6
	35–44	23	24.
	45–54	8	8.3
	55 and over	4	4.2
Education level	Provisional pharmacist	38	39.6
	Prescription pharmacist	47	49
	Under pharmacy education	11	11
	Other	0	0
Educational institution	UiT	3	3.1
	OsloMet	13	13.5
	NTNU	6	6.3
	UiB	4	4.2
	Nord	3	3.1
	UiO	8	8.3
	Fully qualified pharmacist	52	54.2
	Fully trained pharmacist from abroad	7	7.3
Workplace	Pharmacy	88	91.7
	Industry	2	2.1
	Organization	0	0
	Other	6	6.3
Experience	Under 1 year	20	20.8
	1–3 years	15	15.6
	3–6 years	19	19.8
	6–9 years	12	12.5
	10 years and over	30	31.3
District in Norway	East	54	56.3
	West	17	17.7
	North	10	10.4
	Trøndelag	12	12.5
	South	3	3.1
Position	Full time	64	66.7
	Part-time	7	7.3
	On-call substitute	25	26
Customer relations	Very good	47	49
	Good	43	44.8
	Neutral	6	6.3
	Weak	0	0
	Very weak	0	0

**Table 4:** Competence questions and the obtained average scores.

Competence Question	Average Score (Out of 5)
How well are you familiar with social prescribing?	1.98
I believe that social prescribing can be a good way to reach the healthcare system.	3.74
I believe that pharmacists and the pharmacy can have a role in social prescribing.	3.76
I believe that social prescribing can be useful for the individuals who use the offer.	3.97
I believe that pharmaceutical involvement in social prescribing will be useful for the individuals who make use of the offer.	3.81
How confident are you that you are able to identify people who could benefit from social prescribing?	3.14

**Table 5:** Analysis of null hypotheses, statistical tests, and results for competence within community referral across educational levels and genders.

Hypothesis H0:	Statistical Analysis	Result
H0 = There is no significant relationship between competence in social prescribing or community referral and experience in the pharmacy profession	Linear regression	No apparent association
H0 = There is no significant difference in competence about social prescribing or community referral between pharmacists, prescription pharmacists and students	Analysis of variance	No significant difference
H0 = There is no significant difference between competence within social prescribing or community referral and gender	Independent *t*-test	No significant difference

## Data Availability

Data are contained within the article and Supplementary Materials.

## Supplementary Materials

The following supporting information can be downloaded at: https://www.mdpi.com/article/10.3390/pharmacy12020043/s1, The original Norwegian questionnaire and the English translation.

## References

[1] Hough K., Kotwal A.A., Boyd C., Tha S.H., Perissinotto C. (2023). What Are “Social Prescriptions” and How Should They Be Integrated into Care Plans?. AMA J. Ethics.

[2] Oster C., Skelton C., Leibbrandt R., Hines S., Bonevski B. (2023). Models of social prescribing to address non-medical needs in adults: A scoping review. BMC Health Serv. Res..

[3] Husk K., Elston J., Gradinger F., Callaghan L., Asthana S. (2019). Social prescribing: Where is the evidence?. Br. J. Gen. Pract..

[4] Hassan S.M., Ring A., Goodall M., Abba K., Gabbay M., van Ginneken N. (2023). Social prescribing practices and learning across the North West Coast region: Essential elements and key challenges to implementing effective and sustainable social prescribing services. BMC Health Serv. Res..

[5] Vidovic D., Reinhardt G.Y., Hammerton C. (2021). Can Social Prescribing Foster Individual and Community Well-Being? A Systematic Review of the Evidence. Int. J. Environ. Res. Public Health.

[6] Turk A., Tierney S., Wong G., Todd J., Chatterjee H.J., Mahtani K.R. (2022). Self-growth, wellbeing and volunteering—Implications for social prescribing: A qualitative study. SSM—Qual. Res. Health.

[7] Andermann A. (2016). Taking action on the social determinants of health in clinical practice: A framework for health professionals. Cmaj.

[8] Islam K.F., Awal A., Mazumder H., Munni U.R., Majumder K., Afroz K., Tabassum M.N., Hossain M.M. (2023). Social cognitive theory-based health promotion in primary care practice: A scoping review. Heliyon.

[9] Bild E., Pachana N.A. (2022). Social prescribing: A narrative review of how community engagement can improve wellbeing in later life. J. Community Appl. Soc. Psychol..

[10] Morse D.F., Sandhu S., Mulligan K., Tierney S., Polley M., Chiva Giurca B., Slade S., Dias S., Mahtani K.R., Wells L. (2022). Global developments in social prescribing. BMJ Glob. Health.

[11] Chatterjee H.J., Camic P.M., Lockyer B., Thomson L.J.M. (2018). Non-clinical community interventions: A systematised review of social prescribing schemes. Arts Health.

[12] Islam M.M. (2020). Social Prescribing-An Effort to Apply a Common Knowledge: Impelling Forces and Challenges. Front. Public Health.

[13] Kilgarriff-Foster A., O’Cathain A. (2015). Exploring the components and impact of social prescribing. J. Public Ment. Health.

[14] Cooper M., Flynn D., Avery L., Ashley K., Jordan C., Errington L., Scott J. (2023). Service user perspectives on social prescribing services for mental health in the UK: A systematic review. Perspect. Public Health.

[15] Davis-Hall M. (2018). The Bromley by Bow Centre: Harnessing the power of community. Br. J. Gen. Pract..

[16] Leavell M.A., Leiferman J.A., Gascon M., Braddick F., Gonzalez J.C., Litt J.S. (2019). Nature-Based Social Prescribing in Urban Settings to Improve Social Connectedness and Mental Well-being: A Review. Curr. Environ. Health Rep..

[17] Callard F., Friedli L. (2005). Imagine East Greenwich: Evaluating the impact of the arts on health and well-being. J. Public Ment. Health.

[18] Chen I., Opiyo N., Tavender E., Mortazhejri S., Rader T., Petkovic J., Yogasingam S., Taljaard M., Agarwal S., Laopaiboon M. (2018). Non-clinical interventions for reducing unnecessary caesarean section. Cochrane Database Syst. Rev..

[19] Mohiuddin A.K. (2020). The Excellence of Pharmacy Practice. Innov. Pharm..

[20] Katoue M.G., Schwinghammer T.L. (2020). Competency-based education in pharmacy: A review of its development, applications, and challenges. J. Eval. Clin. Pract..

[21] Allayla T.H., Nouri A.I., Hassali M.A.A. (2018). Pharmacist Role in Global Health: A Review of Literature. Malays. J. Pharm. Sci..

[22] Ilardo M.L., Speciale A. (2020). The Community Pharmacist: Perceived Barriers and Patient-Centered Care Communication. Int. J. Environ. Res. Public Health.

[23] Piquer-Martinez C., Urionagüena A., Benrimoj S.I., Calvo B., Martinez-Martinez F., Fernandez-Llimos F., Garcia-Cardenas V., Gastelurrutia M.A. (2022). Integration of community pharmacy in primary health care: The challenge. Res. Soc. Adm. Pharm..

[24] Taylor D.A., Nicholls G.M., Taylor A.D.J. (2019). Perceptions of Pharmacy Involvement in Social Prescribing Pathways in England, Scotland and Wales. Pharmacy.

[25] Lindsey L., Hughes S., Rathbone A. (2021). Social prescribing in community pharmacy: A systematic review and thematic synthesis. Pharm. J..

[26] Kors R. Røde Kors Sosial Resept. https://www.rodekors.no/lokalforeninger/hordaland/om/aktiviteter/sosial-resept/.

[27] Majchrowska A., Bogusz R., Nowakowska L., Pawlikowski J., Piątkowski W., Wiechetek M. (2019). Public Perception of the Range of Roles Played by Professional Pharmacists. Int. J. Environ. Res. Public Health.

[28] Shirdel A., Pourreza A., Daemi A., Ahmadi B. (2021). Health-promoting services provided in pharmacies: A systematic review. J. Educ. Health Promot..

[29] Nelson N.R., Armistead L.T., Blanchard C.M., Rhoney D.H. (2021). The pharmacist’s professional identity: Preventing, identifying, and managing medication therapy problems as the medication specialist. JACCP J. Am. Coll. Clin. Pharm..

[30] Lott B.E., Anderson E.J., Villa Zapata L., Cooley J., Forbes S., Taylor A.M., Manygoats T., Warholak T. (2021). Expanding pharmacists’ roles: Pharmacists’ perspectives on barriers and facilitators to collaborative practice. J. Am. Pharm. Assoc..

[31] Janzen D., Fitzpatrick K., Jensen K., Suveges L. (2013). Women in pharmacy: A preliminary study of the attitudes and beliefs of pharmacy students. Can. Pharm. J..

[32] Wu M.-J., Zhao K., Fils-Aime F. (2022). Response rates of online surveys in published research: A meta-analysis. Comput. Hum. Behav. Rep..

[33] Lee H., Koh S.B., Jo H.S., Lee T.H., Nam H.K., Zhao B., Lim S., Lim J.A., Lee H.H., Hwang Y.S. (2023). Global Trends in Social Prescribing: Web-Based Crawling Approach. J. Med. Internet Res..

[34] Duffull S.B., Wright D.F.B., Marra C.A., Anakin M.G. (2018). A philosophical framework for pharmacy in the 21st century guided by ethical principles. Res. Soc. Adm. Pharm..

[35] McDermott I., Astbury J., Jacobs S., Willis S., Hindi A., Seston E., Schafheutle E. (2023). To be or not to be: The identity work of pharmacists as clinicians. Sociol. Health Illn..

[36] Dinh J.V., Traylor A.M., Kilcullen M.P., Perez J.A., Schweissing E.J., Venkatesh A., Salas E. (2019). Cross-Disciplinary Care: A Systematic Review on Teamwork Processes in Health Care. Small Group Res..

[37] Daly C.J., Quinn B., Mak A., Jacobs D.M. (2020). Community Pharmacists’ Perceptions of Patient Care Services within an Enhanced Service Network. Pharmacy.

[38] Hayhoe B., Cespedes J.A., Foley K., Majeed A., Ruzangi J., Greenfield G. (2019). Impact of integrating pharmacists into primary care teams on health systems indicators: A systematic review. Br. J. Gen. Pract..

[39] Hussein T., Cartright N., Kirschner J., Nadarasa A., Rathbone A.P., Lindsey L. (2024). Social prescribing in pharmacies: What is it, does it work and what does it mean for Canadian pharmacies?. Can. Pharm. J..

[40] Thomas G., Lynch M., Spencer L.H. (2021). A Systematic Review to Examine the Evidence in Developing Social Prescribing Interventions That Apply a Co-Productive, Co-Designed Approach to Improve Well-Being Outcomes in a Community Setting. Int. J. Environ. Res. Public Health.

