# Supplementary material for: Social Prescribing Competence among Community Pharmacists and Pharmacy Students in Norway
Source: Pharmacy (Basel). 2024 Mar 1;12(2):43. doi: 10.3390/pharmacy12020043 (PMC10961744; doi:10.3390/pharmacy12020043)
Supplement: Supplementary file 1 [file pharmacy-12-00043-s001.zip › pharmacy-2848477-supplementary.pdf]

Original Norwegian Online Questionnaire

Nettskjema

Status på kompetanse innen soscial prescribing

(samfunnshenvisning) hos farmasøyter

Status på kompetanse innen soscial prescribing (samfunnshenvisning) hos farmasøyter

Hei!

Vi er en gruppe på 4 farmasi studenter som skriver bacheloroppgave om "social prescribing". Målet med denne studien er å identifisere potensielle roller til farmasøyter ved social prescribing (samfunnshenvisning) og hvordan farmasøyter som en viktig del av helsevesenet kan bidra til å forbedre livskvalitet i det norske samfunnet eller hjelp til å etablere et mer integrert helsesystem i Norge

I forbindelse med dette har vi laget en kort nettbasert spørreundersøkelse som vil ta ca. 5-7 minutter å svare på. Undersøkelsen vil være helt anonym, svarene vil bli analysert og brukt til akademiske formål i vår bacheloroppgave. Ingen personlige eller sensitive data samles inn. Ved å svare på spørsmålene gir du automatisk samtykke til at vi kan bruke resultatene til dette formålet. Denne undersøkelsen består av to deler. Den første delen består av generelt demografiske spørsmål om vedkommende som svarer, og andre delen består av kandidatens formeninger innenfor dette temaet. Vi setter pris på at flest mulig farmasøyter, og eventuelt farmasistudenter som jobber på apotek kan ta seg tiden til å svare på disse spørsmålene.

Hvis du mottar dette spørreskjemaet fra ulike kanaler, vennligst svar på det bare én gang.

Takk på forhånd!

Med vennlig hilsen,

Salia Amir, Siedra Salih, Riyaan Gabeyre og Misbah Hussain

Kun ett mulig svaralternativ, hvis ikke annet er oppgitt.

DEL 1: GENERELLE DEMOGRAFISKE SPØRSMÅL

1. Kjønn

- Mann
- Kvinne
- Annet
- Ønsker ikke å svare

2. Alder (år)

- 18-24
- 25-34
- 35-44
- 45-54
- 55 og over

3. Hva slags type utdanning?

- Provisorfarmasøyt
- Reseptarfarmasøyt
- Fortsatt under farmasi-utdanning
- Annet

3.1. Hvis annet, hva da?

4. Hvis du er student, hvilken utdanningsinstitusjon?

- UIT - Universitetet i Tromsø
- OsloMet - Storbyuniversitetet
- NTNU - Norges teknologiske Naturvitenskapelige Universitetet
- UIB - Universitetet I Bergen
- Nord Universitet
- UiO - Universitet i Oslo
- Ferdigutdannet farmasøyt
- Ferdigutdannet farnasøyt fra utlandet

5. Hvor jobber du?

- Apotek
- Industri
- Firma
- Annet

5.1. Hvis annet, hvor?

6. Hvor lenge har du jobbet som farmasøyt?

- Under 1 år
- 1-3 år
- 3-6 år
- 6-9 år
- 10 år og over

7. Hvilket distrikt i Norge jobber du i?

- Østlandet
- Vestlandet
- Nord-Norge
- Trøndelag
- Sørlandet

8. Hva slags stilling har du?

- Fulltid
- Deltid
- Tilkallingsvikar

9. Hvor god kunderelasjon har du?

- Svært godt
- Godt
- Nøytralt

DEL 2: SOCIAL PRESCRIBING

I denne delen av spørreskjema er det samlet en del kunnskapsspørsmål innen social prescribing (samfunnshenvisning).

Social prescribing (samfunnshenvisning) er en prosess hvor helsepersonell kan referere pasienter til ulike tjenester i samfunnet, ofte er disse gruppene støttet av kommunen eller av frivillige organisasjoner. Dette kan blant annet inkludere ulike støttegrupper, aktiviteter og diverse kurs. Vi ønsker å vite hva du kan, og hva du synes om tema. Svaralternativene vil variere mellom ja/nei, skala fra 1-5 (1 er lavest og 5 er høyest) og flervalgsspørsmål.

Del 2 i): Generell kunnskap om tema

10. Er du kjent med begrepet social prescribing (samfunnshenvisning)?

- Ja
- Nei
- Usikker
- Ønsker ikke å svare

11. Hvor godt er du kjent med social prescribing (samfunnshenvisning)?

- 5. Svært godt
- 4. Godt
- 3. Nøytralt
- 2. Dårlig
- 1. Svært dårlig
- Ønsker ikke å svare

12. Har du brukt social prescribing (samfunnshenvisning) i arbeidsdagen din?

- Ja
- Nei
- Usikker

13. Hvis ja, har pasienten hatt positivt utbytte av social prescribing (samfunnshenvisning)?

- Ja
- Nei
- Usikker

14. Hvis nei, kan du se for deg å prøve ulike tilbud under social prescribing (samfunnshenvisning) i fremtiden?

- Ja
- Nei

Del 2 ii): Identifikasjon av eventuelle endringer og barrierer

15. Kunne du tenkt deg å få større ansvar for social prescribing (samfunnshenvisning) av pasienter som en farmsøyt?

- Ja
- Ingen formening
- Nei

16. Dersom du svarte nei, hvorfor?

- Farmasøyter har nok av andre arbeidsoppgaver
- For lite kunnskap om tema
- Vanskelig å identifisere pasientens behov
- Annet

16.1. Hvis annet, hva?

17. Dersom du svarte ja, hvorfor?

- Mindre belastning på andre helsesektorer
- Tilrettelagt hjelp for pasientene
- Lavterskeltilbud
- Annet

17.1. Hvis annet, hva?

18. I hvilke grad er du enig eller uenig i følgende utsagn?

18.1. Jeg mener at social prescribing kan være en god måte å nå frem til helsevesenet på

- 5. Svært enig
- 4. Enig
- 3. Verken enig eller uenig
- 2. Uenig
- 1. Svært uenig

18.2. Jeg mener at farmasøyter og apoteket kan ha en rolle i social prescribing

- 5. Svært enig
- 4. Enig
- 3. Verken enig eller uenig
- 2. Uenig
- 1. Svært uenig

18.3. Jeg mener at social prescribing kan være nyttig for individene som benytter seg av tilbudet

- 5. Svært enig
- 4. Enig
- 3. Verken enig eller uenig
- 2. Uenig
- 1. Svært uenig

18.4. Jeg mener at farmasøytisk involering i social prescribing vil være nyttig for individene som benytter seg av tilbudet

- 5. Svært enig
- 4. Enig
- 3. Verken enig eller uenig
- 2. Uenig
- 1. Svært uenig

19. Hvor trygg er du på at du er i stand til å identifisere mennesker som kan ha nytte av social prescribing (samfunnshenvisning)?

- 5. Svært trygg
- 4. Trygg
- 3. Verken trygg eller utrygg
- 2. Utrygg
- 1. Svært utrygg

20. Som en farmasøyt, føler du deg trygg nok til å kunne identifisere mennesker som kan få nytte av ikke-kliniske tilbud eller tiltak

- Ja
- Nei
- Usikker

21. Om du skal bli mer involvert i social prescribing (samfunnshenvisning), hva slags opplæring mener du at du behøver for å kunne utføre den rollen?

Flere mulige svaralternativer kan velges

- Forstå mer om social prescribing (samfunnshenvisning)
- Forstå mer om farmasøytens rolle i social prescribing (samfunnshenvisning)
- Bedre kommunikasjonsevner
- Vite mer om hvilke aktiviteter som er tilgjengelig
- Inkluderingskriterier for aktivitetene/tiltak eller tilbud
- Tilstandsspesifikk informasjon, for eksempel symptomer og tegn
- Annet

21.1. Hvis annet, hva da?

22. Hvilke av disse eksemplene for social prescribing (samfunnshenvisning) kunne du tenkt deg å henvise til som farmasøyt?

Flere mulige svaralternativer kan velges

- Meditasjon eller yoga
- Utendørsaktiviteter
- Biblioterapi (selvhjelpsbøker)
- Kunst aktiviteter
- Livsstilssendring / kostholdsråd

- Annet

22.1. Hvis annet, hva da?

23. Er det noe annet du ønsker å legge til?

Translated version of the questionnaire

Online form

Status of competence in social prescribing (community referral) among pharmacists

Hello!

We are a group of 4 pharmacy students who are writing a bachelor's thesis on "social prescribing". This study aims to identify potential roles for pharmacists in social prescribing (community referral) and how pharmacists as an important part of the healthcare system can contribute to improving the quality of life in Norwegian society or help to establish a more integrated healthcare system in Norway.

In connection with this, we have created a short online survey, which will take approx. 5-7 minutes to answer. The survey will be completely anonymous; the answers will be analyzed and used for academic purposes in our bachelor thesis. No personal or sensitive data is collected. By answering the questions, you automatically give consent for us to use the results for this purpose. This survey consists of two parts. The first part consists of general demographic questions about the respondent, and the second part consists of the candidate's thoughts on this theme. We appreciate that as many pharmacists as possible, and possibly pharmacy students who work in a pharmacy, can take the time to answer these questions.

If you receive this questionnaire from different channels, please answer it just once. Thanks in advance!

Sincerely,

Salia Amir, Siedra Salih, Riyaan Gabeyre and Misbah Hussain

Only one possible answer alternative, unless otherwise stated.

PART 1: GENERAL DEMOGRAPHIC INFORMATION

1. Gender

- Man
- Woman
- Other
- Don't want to answer

2. Age (years)

- 18-24
- 25-34
- 35-44
- 45-54
- 55 and over

3. What type of education?

- Provisional pharmacist
- Prescription pharmacist
- Still undergoing pharmacy education
- Other

3.1. If not, then what?

4. If you are a student, which educational institution?

- UIT - University of Tromsø
- OsloMet - Storbyuniversitetet
- NTNU - The Norwegian University of Technological Sciences
- UIB - University of Bergen
- North University
- UiO - University of Oslo
- Fully qualified pharmacist
- Pharmacist from abroad

5. Where do you work?

- Drugstore
- Industry
- Business
- Other

5.1. If other, where?

6. How long have you worked as a pharmacist?

- under 1 year
- 1-3 years
- 3-6 years
- 6-9 years
- 10 years and over

7. Which district in Norway do you work in?

- Eastland
- Westland
- Northern Norway
- Trøndelag
- Southland

8. What kind of position do you have?

- Full time
- Part-time
- On-call substitute

9. How good is your customer relationship?

- Very good
- Well
- Neutral

PART 2: SOCIAL PRESCRIBING

In this part of the questionnaire, several knowledge questions about social prescribing (community referral) have been collected.

Social prescribing (community referral) is a process where healthcare personnel can refer patients to various services in society, often these groups are supported by the municipality or by voluntary organizations. This can include, among other things, various support groups, activities, and various courses. We want to know what you can do and what you think about the theme. The answer alternatives will vary between yes/no, scale from 1-5 (1 is the lowest and 5 is the highest), and multiple choice questions.

Part 2 i): General knowledge of the theme

10. Are you familiar with the term social prescribing?

- Yes
- No
- Insecure
- Don't want to answer

11. How well are you familiar with social prescribing?

- 5. Very good
- 4. Good
- 3. Neutral
- 2. Bad
- 1. Very bad
- Don't want to answer

12. Have you used social prescribing in your working day?

- Yes
- No
- Not sure

13. If yes, has the patient benefited positively from social prescribing (community referral)?

- Yes
- No
- Insecure

14. If not, can you imagine trying different offers under social prescribing in the future?

- Yes
- No

Part 2 ii): Identification of any changes and barriers

15. Could you imagine having greater responsibility for social prescribing (community referral) of patients as a farm assistant?

- Yes

- No idea
- No

16. If you answered no, why?

- Pharmacists have enough other tasks
- Too little knowledge of the topic
- Difficult to identify the patient's needs
- Other

16.1. If else, what?

17. If you answered yes, why?

- Less burden on other health sectors
- Organized help for the patients
- Low threshold offer
- Other

17.1. If else, what?

18. To what extent do you agree or disagree with the following statements?

18.1. I believe that social prescribing can be a good way to reach the healthcare system

- 5. Strongly agree
- 4. Agree
- 3. Neither agree nor disagree
- 2. Disagree
- 1. Strongly disagree

18.2. I believe that pharmacists and the pharmacy can have a role in social prescribing

- 5. Strongly agree
- 4. Agree
- 3. Neither agree nor disagree
- 2. Disagree
- 1. Strongly disagree

18.3. I believe that social prescribing can be useful for the individuals who make use of the offer

- 5. Strongly agree
- 4. Agree
- 3. Neither agree nor disagree
- 2. Disagree
- 1. Strongly disagree

18.4. I believe that pharmaceutical involvement in social prescribing will be useful for the individuals who make use of the offer

- 5. Strongly agree
- 4. Agree

- 3. Neither agree nor disagree
- 2. Disagree
- 1. Strongly disagree

19. How confident are you that you can identify people who can benefit from social prescribing?

- 5. Very safe
- 4. Safe
- 3. Neither safe nor unsafe
- 2. Unsafe
- 1. Very unsafe

20. As a pharmacist, do you feel confident enough to be able to identify people who may benefit from non-clinical offers or interventions

- Yes
- No
- Insecure

21. If you are to become more involved in social prescribing (community referral), what kind of training do you think you need to be able to perform that role?

Several possible answer alternatives can be selected

- Understand more about social prescribing
- Understand more about the pharmacist's role in social prescribing (community referral)
- Better communication skills
- Find out more about which activities are available
- Inclusion criteria for the activities/initiatives or offers
- Condition-specific information, for example, symptoms and signs
- Other

21.1. If other, then what?

22. Which of these examples of social prescribing (community referral) could you imagine referring to as a pharmacist?

Several possible answer alternatives can be selected

- Meditation or yoga
- Outdoor activities
- Bibliotherapy (self-help books)
- Art activities
- Lifestyle change / dietary advice
- Other

22.1. If other, then what?

23. Is there anything else you would like to add?
